# The Effect of the Fresh Latex Ratio on the Composition and Properties of Bio-Coagulated Natural Rubber

**DOI:** 10.3390/polym17162211

**Published:** 2025-08-13

**Authors:** Jianwei Li, Honghai Huang, Li Ding, Tuo Dai, Haoran Geng, Tao Zhao, Liguang Zhao, Fan Wu, Hongxing Gui

**Affiliations:** 1 Rubber Research Institute, Chinese Academy of Tropical Agricultural Sciences, Haikou 571101, China; 18272733025@163.com (J.L.); honghaihuang2009@163.com (H.H.); gdzjding@163.com (L.D.); catasdaituo@163.com (T.D.); haorangeng@whut.edu.cn (H.G.); m15595730862@163.com (T.Z.); zhaoliguang@catas.com (L.Z.); crucxjsdjd@163.com (F.W.); 2 Hainan Provincial Special Natural Rubber Processing Technology Innovation Center, Haikou 571101, China; 3 Center for Smart Materials and Devices, State Key Laboratory of Advanced Technology for Materials Synthesis and Processing, Wuhan University of Technology, Wuhan 430070, China

**Keywords:** fresh latex, composition, performance, bio-coagulated, natural rubber

## Abstract

By proportionally blending fresh latex from PR107, Reyan 72059, and Reyan 73397, and employing both acid- and enzyme-assisted microbial coagulation methods, this study analyzed the effects of the specific latex formulation on the following: physicochemical properties, non-rubber components, molecular weight and distribution, vulcanization characteristics of compounded rubber, and physical–mechanical properties of vulcanized natural rubber. The results indicate that, compared to acid-coagulated natural rubber, enzyme-assisted microbial coagulated natural rubber exhibits slightly lower levels of volatile matter, impurities, plasticity retention index (PRI), nitrogen content, calcium ions (Ca^2+^), iron ions (Fe^3+^), and fatty acid content. Conversely, it demonstrates higher values in ash content, initial plasticity (P_0_), Mooney viscosity (M_L(1+4)_), acetone extract, magnesium ions (Mg^2+^), copper ions (Cu^2+^), manganese ions (Mn^2+^), gel content, molecular weight and distribution, and glass transition temperature (Tg). With the increase in the proportion of PR107 and Reyan 72059 fresh latex, the ash content, volatile matter content, fatty acid content, gel content, and dispersion coefficient (PDI) of natural rubber gradually decrease, while the impurity content, PRI, nitrogen content, weight-average molecular weight (Mw), and number-average molecular weight (Mn) gradually increase. Compared to acid-coagulated natural rubber compounds, enzyme-assisted microbial-coagulated natural rubber compounds exhibit higher minimum torque (M_L_) and maximum torque (M_H_), but shorter scorch time (t_10_) and optimum cure time (t_90_). Furthermore, as the proportion of PR107 and Reyan 72059 fresh latex increases, the ML of the compounds gradually decreases. In pure rubber formulations, enzyme-assisted microbial-coagulated natural rubber vulcanizates demonstrate higher tensile strength, tear strength, modulus at 300%, and Shore A hardness compared to acid-coagulated natural rubber vulcanizates. When the fresh latex ratio of PR107, Reyan 72059, and Reyan 73397 is 1:1:3, the tensile strength and 300% modulus of the natural rubber vulcanizates reach their maximum values. In carbon black formulations, the tensile strength and tear strength of enzyme-assisted microbial-coagulated natural rubber vulcanizates are significantly higher than those of acid-coagulated natural rubber vulcanizates in pure rubber formulations, with the increase exceeding that of other samples.

## 1. Introduction

Natural rubber (NR) is a kind of elastic polymer material processed from a rubber tree biosynthesis of milky white liquid (fresh latex). It has excellent comprehensive performance (high strength, high elasticity, high abrasion resistance, etc.) and is widely used in the fields of national defense, military industry, industry, agriculture, and health care, and is therefore closely related to national security, economic construction, and people’s lives [1,2]. The main components of fresh latex are cis-1,4-polyisoprene and water, along with small amounts of non-rubber components (such as proteins, acetone-soluble substances, inorganic salts, etc.). The composition and properties of natural rubber produced from fresh latex are influenced by various agricultural factors (such as variety, soil, season, climate), among which the rubber tree variety plays a crucial role [3,4,5,6].

Research conducted by scholars both domestically and internationally has revealed significant variations in the composition, structure, and properties of natural rubber derived from fresh latex produced by different varieties of rubber trees. Bonfils et al. [7] characterized the microstructures of RRIM600, GT1, RRIC110, and PB217 natural rubbers and found that PB217 natural rubber contained heavier and denser micro-aggregates than the other varieties. Wisunthorn et al. [8] studied the dynamic structure of the RRIM600 and PB235 varieties of NR using SEC-MALS and found that the rapid structuring of the two NRs resulted in a significant increase in macromolecular structure, especially in the average molecular weight (Mn) and gel content. Simin et al. [6] analyzed the relationship between particle size and natural rubber properties of natural latexes from IAN873, Dongfang 93114, and Reyan 73397, and found that small rubber particles had longer molecular chains, and large rubber particles had wider molecular weight distributions. The ester content of small rubber particles of IAN873 and Reyan 73397 was almost zero. Lü Feijie et al. [9] analyzed the composition and structure of PR107, PB86, GT1, Haiken 1, and the seedling NR samples, and found that there were significant differences in the composition and content of non-rubber components and molecular structure among different varieties of NR. Wu Cui et al. [10] conducted a comprehensive analysis of the natural rubber properties across six Hevea brasiliensis cultivars, namely RRIM600, Reyan 72059, Reyan 8813, Reyan 73397, PR107, and Reyan 879. The experimental results demonstrated significant variations in both the chemical composition of the fresh latex and the performance characteristics of natural raw rubber among different cultivars. Lehman et al. [11] analyzed the particle size and properties of natural latex from RRIM600, RRIT251, PB235, and BPM24. The results showed that RRIM600 natural latex had a larger particle size and uniform distribution and exhibited good mechanical properties compared to other varieties.

However, this study focuses on the main varieties of rubber available in the Hainan region: PR107, Reyan 72059 and Reyan 73397 rubber tree fresh latex. It provides a preliminary exploration and analysis of these three varieties of fresh latex mixed in certain proportions and coagulated by biological and other methods. The study compares the composition and performance of the resulting natural rubber. 

This study provides a theoretical basis for the quality control of high-performance natural rubber, helps to break through the bottleneck of high-performance processing technology of domestic natural rubber, improves the comprehensive performance of domestic natural rubber, and promotes the localization process of natural rubber for high-end products in China.

## 2. Materials and Methods

### 2.1. Materials and Instruments

The fresh latex samples of PR107, Reyan 72059 (dry rubber content of 36.5%), and Reyan 73397 (dry rubber content of 35.0%), along with microbial strains (lactic acid bacteria), were provided by the Rubber Research Institute, Chinese Academy of Tropical Agricultural Science. Alkaline protease (200,000 U/g) was supplied by Beijing Coolab Technology Co., Ltd. (Beijing, China) Formic acid, zinc oxide, stearic acid, sulfur, and N330 carbon black were provided by Sinopharm Chemical Reagent Co., Ltd. (Shanghai, China).

The equipment was sourced as follows: Electronic balance, Orebo FA2204 B. Rotorless rheometer, Goodtechwill testing machines (Qingdao) Co., Ltd. (Qingdao, China) M-3000A. Gel Permeation Chromatography (GPC), Shanghai Sunflower Electronic Technology Co., Ltd. (Shanghai, China) Waters 1515. Open-type rubber mixing machine, Guangdong Zhanjiang Machinery Factory JTC-752. Platen vulcanizing press, Guangdong Chaozhou Hongqiao Rubber Machinery Factory (Zhanjiang, China) 250KWXLB-D. Tensile testing machine, Goodtechwill testing machines (Qingdao) Co., Ltd. (Qingdao, China) AI-7000-SGD1.

### 2.2. Methods

(1) Microbial coagulant liquid culture

Mix the microbial strain, molasses, and water in a ratio of 1:5:94 (by mass). Cultivate the microbial gelatin solution at a temperature of 25 to 40 degrees Celsius for 3 to 4 days. The pH value of the microbial gelatin solution is 3.5 to 3.8. Store it for later use.

(2) Natural rubber preparation

Dilute the dry rubber content of fresh latex to 23%. Mix the fresh latex according to the ratio in Table 1. Stir the mixed fresh latex and add the coagulant evenly. After the fresh latex has completely coagulated, cure the coagulated blocks for 2 days. Then, press, wash, hang, and dry them at a low temperature to prepare natural rubber samples, labeled as H-1, H-2, H-3, H-4, and H-5. Single-variety natural rubber coagulated by acid coagulation or enzyme-assisted microbial coagulation is used as the control sample.

(3) Preparation of natural rubber compound

The natural rubber compound was prepared by taking 300 g raw rubber and using a pure rubber formulation (Table 2) according to the GB/T 6038-2006 [12] standard.

The natural rubber compound was prepared by taking 500 g raw rubber and using a carbon black formula (Table 3) according to the NY/T 1403-2007 [13] standard.

(4) Preparation of natural rubber vulcanized rubber

The natural rubber compound was placed in a flat vulcanization machine, and vulcanized test pieces were prepared under the condition of 140 °C for 30 min.

## 3. Testing and Analysis

(1) The P_0_, PRI, M_L(1+4)_, ash content, impurity content, and volatile matter content of natural rubber were determined according to GB/T 3510-2006 [14], GB/T 3517-2014 [15], GB/T 1232.1-2016 [16], GB/T 4498.1-2013 [17], GB/T 8086-2019 [18], and GB/T 24131-2009 [19] standards.
(2) The nitrogen content, acetone-soluble content, fatty acid content, and metal ion content of natural rubber were determined according to GB/T 8088-2008 [20], GB/T 3516-2006 [21], Q/440803 JGS003-2020 [22], and GB/T 39486-2020 [23] standards.
(3) The gel content of natural rubber was determined by the GB/T 37498-2019 [24] standard. The molecular weight was measured using gel permeation chromatography under the following conditions: sample concentration of 2 mg/mL, tetrahydrofuran as the mobile phase, and polystyrene as the standard reference material.
(4) The Tg of natural rubber was determined using a DSC 214 Polyma differential scanning calorimeter under the following test conditions: nitrogen atmosphere (flow rate of 0.05 L/min), cooling from room temperature to −90 °C, followed by heating to 25 °C at a rate of 10 °C/min.
(5) The vulcanization characteristics of natural rubber compounds under pure gum formulations were determined using a closed-cup rheometer. The testing conditions were as follows: a test temperature of 143 °C, a test duration of 45 min, an oscillation frequency of 1.7 Hz, and an amplitude of ±0.5°.
(6) The physical and mechanical properties of the natural rubber vulcanized rubber were determined by a tensile testing machine according to GB/T 528-2009 [25] and GB/T 529-2008 [26] standards, with a running speed of 500 mm/min.

## 4. Results and Analysis

### 4.1. Physical and Chemical Indexes of Natural Rubber

**Figure 1:**
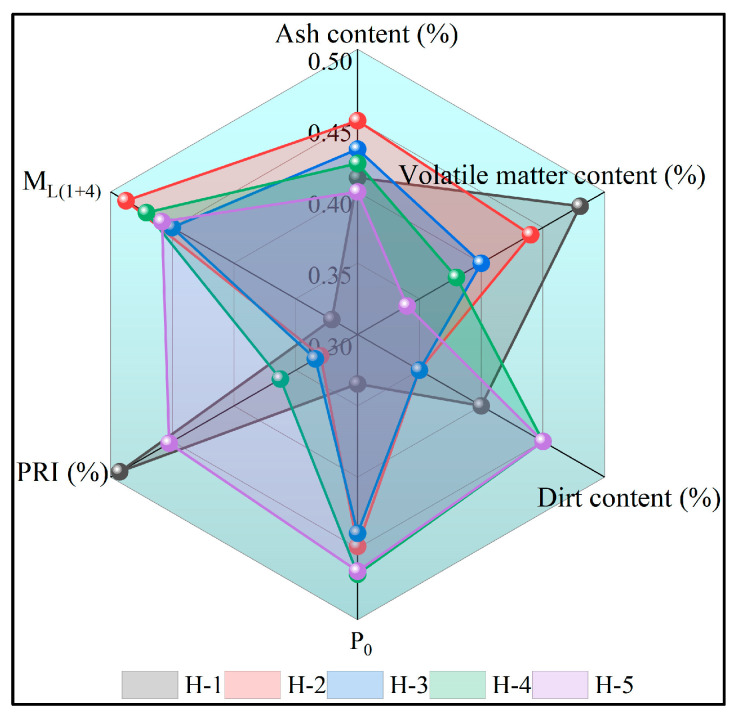
Physical and chemical indexes of natural rubber.

Ash reflects the inorganic salts remaining after the rubber is burned in a high-temperature furnace; volatiles reflect the quality of each 100 g of standard rubber before and after the heating reduction; impurities reflect the latex collection and processing from the outside world mixed with solid substances; P_0_ reflects the degree of rubber plasticity, which is inversely proportional to the plasticity; PRI reflects the strength of the oxidation resistance of the rubber, which is directly proportional to the oxidation resistance; M_L(1+4)_ reflects the processing performance of the rubber, which is inversely proportional to the processing performance [27]. Changes in physical and chemical indicators of natural rubber are presented in Table 4 and Figure 1. It can be seen that, compared with acid coagulation of natural rubber, the enzyme-assisted microbial coagulation of natural rubber results in a slightly lower volatile content, impurity content, and PRI, while the ash content, P_0_, and M_L(1+4)_ are high and in line with the national standards, indicating that the coagulation process has a significant impact on the physicochemical indexes of natural rubber. This is because the coagulation rate during enzyme-assisted microbial coagulation of natural rubber is relatively slow. The degree of crosslinking in the rubber network structure is high, increasing the difficulty of plasticizing natural rubber, and reducing the plasticity of natural rubber [28]. Enzymatic and microbial metabolic processes consume a portion of proteins and other non-rubber components in the latex, leading to a reduction in natural anti-aging constituents and consequently diminishing the oxygen aging resistance of natural rubber [29]. With an increasing proportion of PR107 and Reyan 72059 fresh latex, the ash content and volatile matter content of natural rubber exhibit a slight decrease, while the impurity content and PRI show a marginal increase. Notably, P_0_ and M_L(1+4)_ demonstrate no significant trend of variation. These observations indicate that the inorganic salt content in the mixed fresh latex of PR107 and Reyan 72059 is relatively low, resulting in reduced volatile matter content in the rubber. Furthermore, the introduction of external impurities during rubber processing may contribute to the increased impurity content in natural rubber. The higher concentration of natural anti-aging components in PR107 and Reyan 72059 fresh latex enhances the oxygen aging resistance of natural rubber.

### 4.2. Non-Rubber Components of Natural Rubber

#### 4.2.1. Nitrogen and Acetone Extract Content

**Figure 2:**
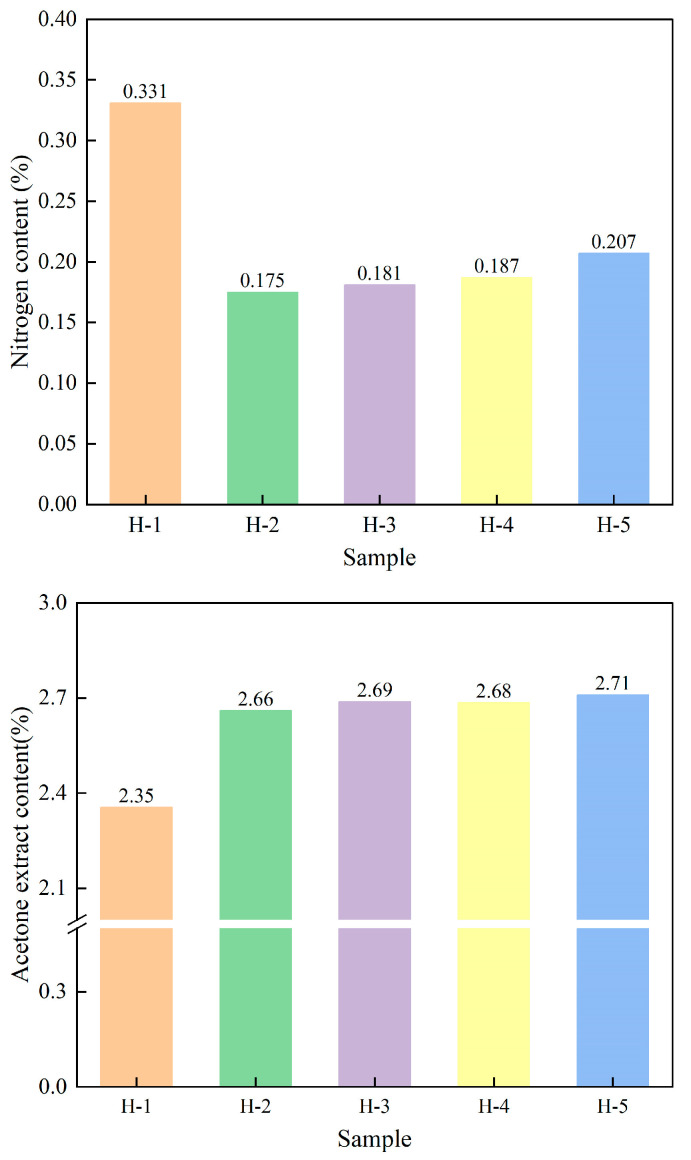
The nitrogen and acetone extract content of natural rubber.

The nitrogen in natural rubber is mainly present in its protein component, accounting for approximately 16% of the protein mass. The protein content can be calculated by measuring the nitrogen content, while the acetone-soluble substances in rubber are collectively referred to as the acetone extract [30]. The changes in the nitrogen content and acetone extract content of natural rubber are shown in Figure 2. As can be seen from the figure, compared with acid coagulation of natural rubber, enzyme-assisted microbial coagulation of natural rubber results in lower nitrogen content and a higher content of acetone extract, indicating that the coagulation process has a significant effect on the content of non-rubber components of natural rubber. Conversely, the addition of acid coagulation results in most of the proteins being retained in the natural rubber. In the case of enzyme-assisted microbial coagulation, the enzymes and microorganisms metabolize some of the proteins and other non-rubber components of the latex, and some of the metabolites of the other non-rubber components are converted to acetone solvents [31,32]. As the ratio of PR107, Reyan 72059 fresh latex increased, the nitrogen content of the natural rubber gradually increased, while the content of acetone extract tended to stabilize, indicating that the protein content of PR107, Reyan 72059 mixed fresh latex is higher. This is because the rubber tree species is one of the key factors affecting the non-gum components of natural rubber [33,34].

#### 4.2.2. Metal Ion Content

**Figure 3:**
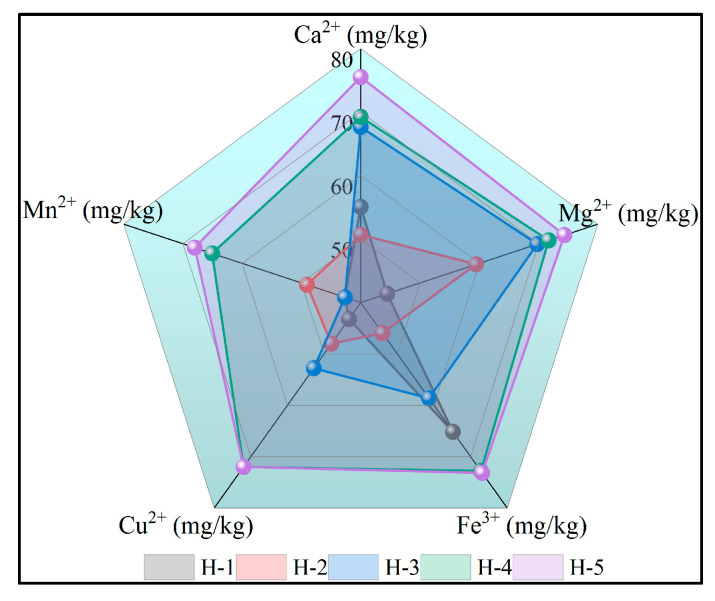
The metal ion content of natural rubber.

The variation in metal ion content in natural rubber is illustrated in Figure 3. As depicted, enzyme-assisted microbial coagulation of natural rubber exhibits higher concentrations of Mg^2+^, Cu^2+^, and Mn^2+^ compared to acid-coagulated natural rubber, while Ca^2+^ and Fe^3+^ levels are relatively lower. This observation suggests that the coagulation process has a significant impact on the metal ion content in natural rubber. The elevated levels of Mg^2+^, Cu^2+^, and Mn^2+^ can be attributed to their role as activators for various enzymes, facilitating their binding to proteases and subsequent retention in the rubber matrix during enzyme-assisted microbial coagulation. Although Ca^2+^ and Fe^3+^ also participate in regulating enzyme activity, their concentrations may be reduced due to complexation or precipitation during the coagulation process, as evidenced in references [35,36]. With the increase of PR107 and Reyan 72059 fresh latex ratios, the Ca^2+^, Mg^2+^, and Cu^2+^ contents of natural rubber were higher than those of control sample H-2, indicating that the Ca^2+^, Mg^2+^, and Cu^2+^ of the mixed fresh latex of PR107 and Reyan 72059 were higher than those of Reyan 73397, which was attributed to the significant effect of the rubber tree species on the metal ion content of fresh latex [37,38].

#### 4.2.3. Fatty Acid Content

**Figure 4:**
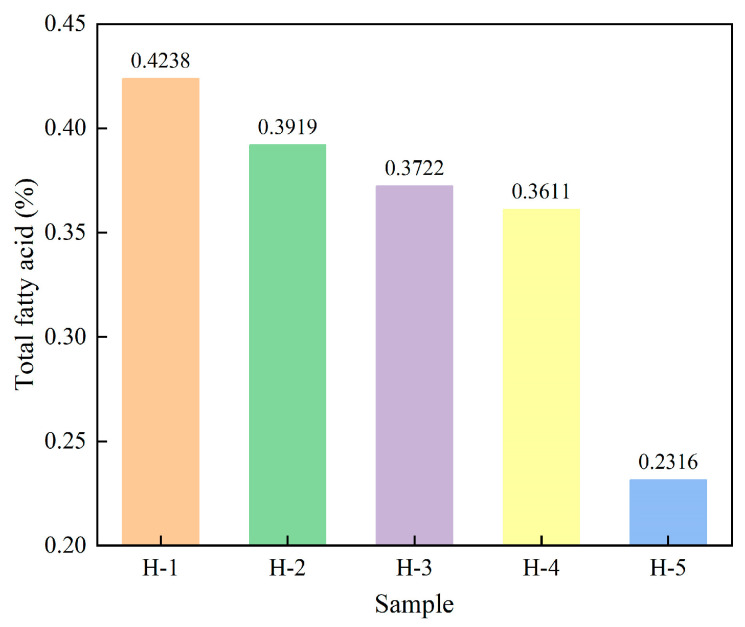
The fatty acid content of natural rubber.

Fatty acids are an important part of the non-rubber component of natural rubber [39]. The fatty acid content of natural rubber varied as illustrated in Figure 4. From the figure, it can be seen that the fatty acid content of enzyme-assisted microbial coagulation of natural rubber was lower compared with that of acid coagulation of natural rubber, indicating that enzyme-assisted microbial coagulation is conducive to the reduction of the fatty acid content of natural rubber. This is because, during the process of enzyme-assisted microbial coagulation, fatty acids not only act as nutrients for microorganisms, but also undergo oxidative reactions with oxygen, which can lead to a reduction in their content [39]. The fatty acid content of enzyme-assisted microbial coagulation of natural rubber gradually decreased with the increase of the ratio of PR107 and Reyan 72059 fresh latex, indicating that the fatty acid content of mixed fresh latex of PR107 and Reyan 72059 was relatively low, which is because the fatty acid content of fresh latex is affected by the rubber tree species [3,4].

### 4.3. Gel Content of Natural Rubber

**Figure 5:**
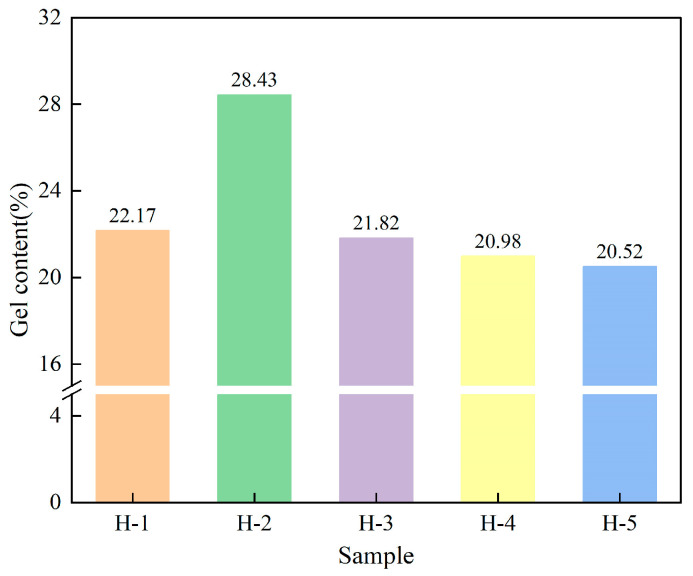
The gel content of natural rubber.

In natural rubber, gel refers to the branched or cross-linked formations of rubber insoluble in organic solvents. Thus, gel reflects the micro “cross-linked” network structure and morphology of natural rubber [31,40]. The variation of the gel content of natural rubber is illustrated in Figure 5. From the figure, it can be seen that the gel content of enzyme-assisted microbial coagulation of natural rubber is higher compared with that of acid coagulation, indicating that the enzyme-assisted microbial coagulation process is conducive to the production of gels. This is due to the relatively high pH environment of enzyme-assisted microbial coagulation and the slow coagulation process, which allows the free radicals in the rubber molecules to be sufficiently cross-linked, whereas acid coagulation impedes this structuring of rubber molecular chains during the ripening process [39,41]. The gel content of natural rubber gradually decreased as the ratio of PR107 and Reyan 72059 fresh latex increased, indicating that the fresh latex variety had a significant effect on the formation of natural rubber gel, which may be attributed to the low content of free radicals in the mixed fresh latexes of PR107 and Reyan 72059, resulting in a low degree of structuring of the rubber molecular chains [42,43], consequently leading to a decrease in the gel content of the natural rubber.

### 4.4. Molecular Weight Size and Distribution of Natural Rubber

**Figure 6:**
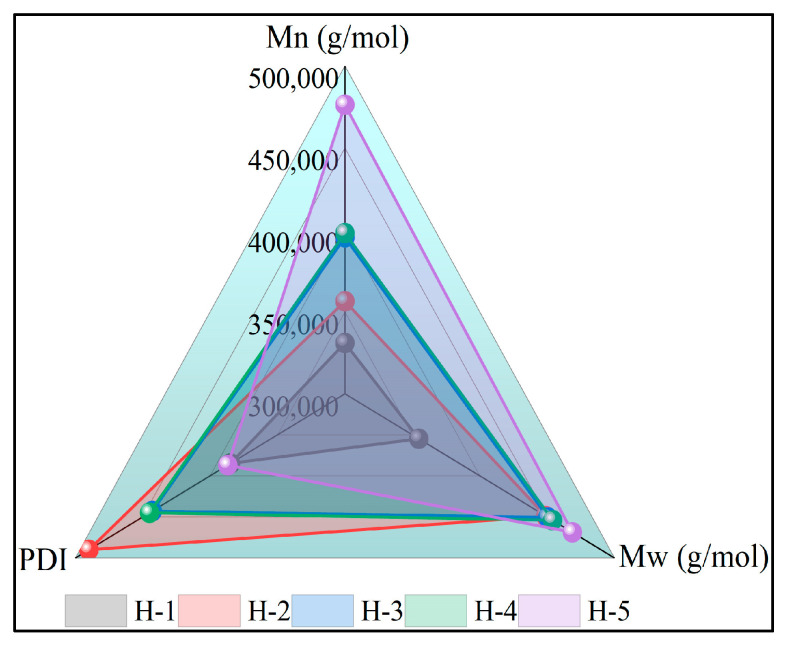
The molecular weight size and distribution of natural rubber.

The low molecular weight portion of natural rubber gives the rubber good plasticity and flowability, while the medium and high molecular weight portions provide good elasticity and mechanical strength [44]. The variation in the size and distribution of natural rubber molecular weights is illustrated in Figure 6. From the figure, it can be seen that the Mw, Mn, and PDI of enzyme-assisted microbial coagulation of natural rubber were larger compared with that of acid-coagulated natural rubber, indicating that the coagulation process has a significant effect on the size and distribution of the molecular weight of natural rubber. This may be because enzyme-assisted microbial coagulation promotes more branched cross-linking of short-chain rubber hydrocarbon molecules during the coagulation process [45]. With the increase of fresh latex ratios of PR107 and Reyan 72059, the Mw and Mn of natural rubber gradually increased, and the PDI gradually decreased, indicating that the fresh latex ratios have a significant effect on the molecular weight size and distribution of natural rubber, which is due to the significant differences in the composition and structure of the fresh latex of different varieties of rubber trees [11,46].

### 4.5. Glass Transition Temperature of Natural Rubber

**Figure 7:**
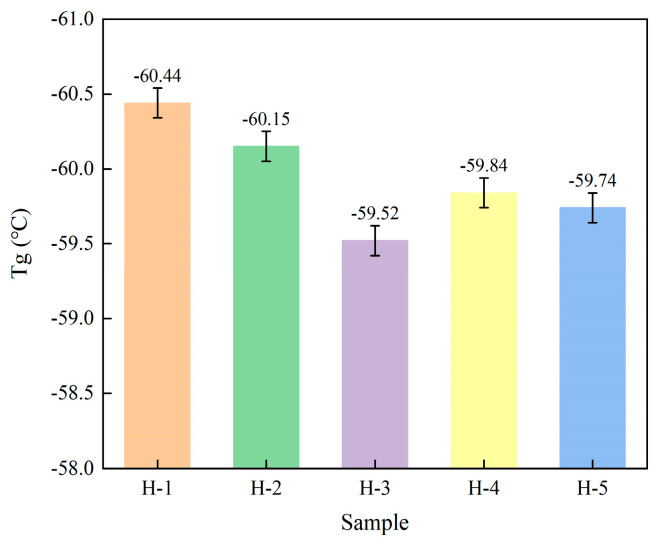
The glass transition temperature of natural rubber.

The Tg is the critical temperature corresponding to the transition between the glassy and highly elastic states of natural rubber. Above Tg, natural rubber exhibits an elastic state; below Tg, natural rubber loses its elasticity and behaves as a rigid solid similar to glass, with less deformation occurring under external forces. The variation in the Tg of natural rubber is shown in Figure 7. From the figure, it can be seen that the Tg of enzyme-assisted microbial coagulation of natural rubber is relatively high compared with that of acid-coagulated natural rubber, and with the addition of PR107 and Reyan 72059 fresh latex, the Tg is also significantly higher than that of acid-coagulated natural rubber, indicating that both the coagulation process and the fresh latex ratio affect the Tg of natural rubber [47].

### 4.6. Vulcanization Characteristics of Natural Rubber Compound

**Figure 8:**
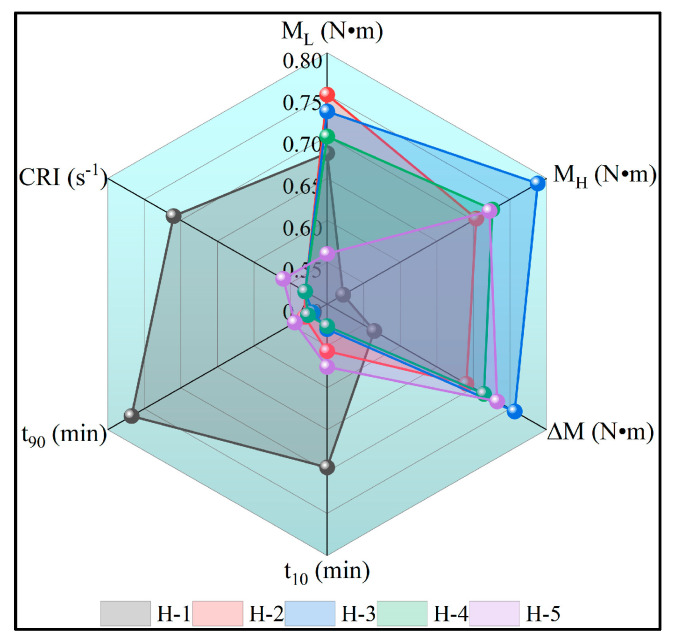
The vulcanization characteristics of natural rubber compounds.

The M_L_ reflects the fluidity of the rubber at a specific temperature, the maximum torque (M_H_) reflects the maximum degree of cross-linking of the rubber, and the difference in torque ∆M reflects the high degree of cross-linking of the rubber; the t_10_ reflects the length of the induced period of vulcanization of the rubber, and the t_90_ reflects the time required for the rubber to reach 90% of the maximum torque [48,49]. The variation in vulcanization characteristics of natural rubber compounds is shown below (Table 5, Figure 8). From the figure, it can be seen that the M_L_ and M_H_ of enzyme-assisted microbial coagulation of natural rubber blends are larger, while t_10_ and t_90_ are smaller compared to acid-coagulated natural rubber blends, indicating that enzyme-assisted microbial coagulation can increase the mobility and maximum cross-linking degree of the natural rubber, accelerating its vulcanization rate while reducing the processing safety. This is because non-gum components such as proteins and acetone solutes may be converted by microorganisms into choline, acetone, and acetone solvates in the process of enzyme-assisted microbial coagulation. They may also be converted to bile ammonia or amino acids. The coordination between these amines and the zinc atoms in the zinc 2,2′-di benzothiazole disulfide (MBTS) complexes improves the solubility of the complexes in the rubber and further enhances the nucleophilicity of sulfur in the complexes towards the S8 ring, which would increase the rate of pre-cure formation and decrease the rate of cross-link shortening [50]. The M_L_ of the natural rubber blends gradually decreased with the increase in the ratio of PR107, Reyan 72059 fresh latex, indicating a gradual decrease in the fluidity of the natural rubber blends. This was attributed to the gradual increase in Mw and Mn of the natural rubber blends and the gradual decrease in PDI, and the enhancement of intermolecular forces between the rubber molecules [31]. The PR107, Reyan 72059, and Reyan 73397 fresh latex ratio of 1:1:3 resulted in the maximum ∆M of the natural rubber blends, indicating the highest degree of cross-linking of the H-3 vulcanized rubber.

### 4.7. Physical and Mechanical Properties of Natural Rubber Vulcanized Rubber

**Figure 9:**
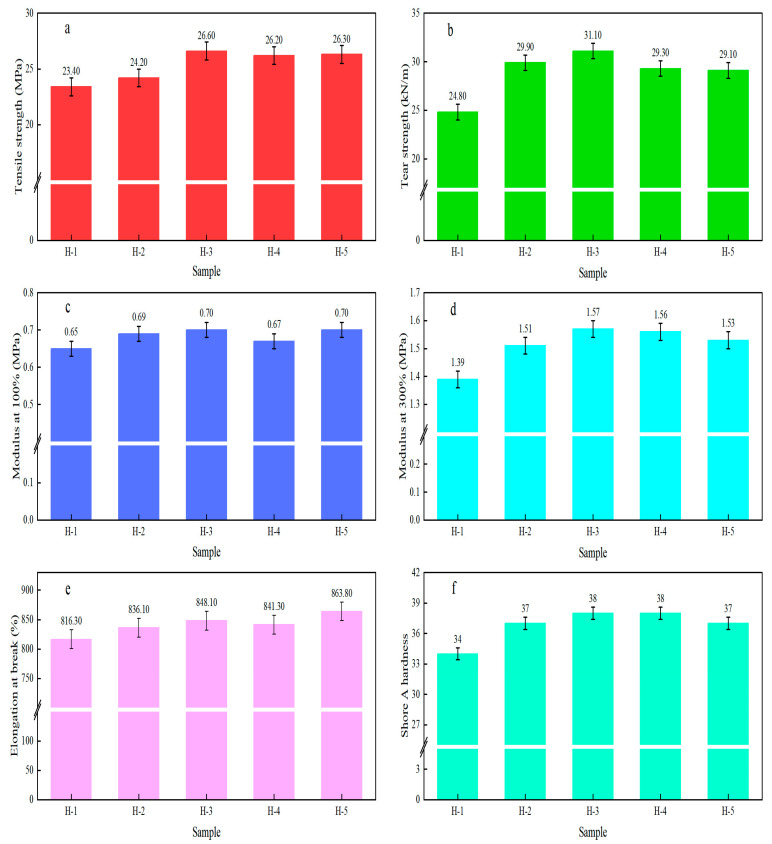
The physical and mechanical properties of natural rubber vulcanized rubber (pure rubber formulation). (**a**) Tensile strength; (**b**) Tearing strength; (**c**) Modulus at 100%; (**d**) Modulus at 300%s; (**e**) Elongation at break; (**f**) Shore A hardness.

The changes in the physical and mechanical properties of natural rubber vulcanized rubber (pure rubber formulation) are illustrated in Figure 9. From the figure, it can be seen that the tensile strength, tear strength, modulus, and shore A hardness of enzyme-assisted microbial coagulation natural rubber vulcanized rubber were larger compared to acid-coagulated natural rubber vulcanized rubber, indicating that the physical and mechanical properties of enzyme-assisted microbial coagulation natural rubber vulcanized rubber were superior in the conditions of the pure rubber formulation, due to the larger ∆M of the enzyme-assisted microbial coagulation natural rubber blends and the high degree of crosslinking [50,51]. The tensile strength, modulus at 300%, and elongation at break of the natural rubber vulcanized rubber increased with the addition of PR107, Reyan 72059 fresh latex, indicating that the mixing of PR107, Reyan 72059, and Reyan 73397 fresh latex had a significant effect on the physical and mechanical properties of the natural rubber vulcanized rubber. When the ratio of PR107, Reyan 72059, and Reyan 73397 fresh latex was 1:1:3, the tensile strength and modulus at 300% of the natural rubber vulcanized rubber reached the maximum values of 26.60 MPa and 1.57 MPa, which were 8.2~9.91% and 1.32~3.97% higher than those of the control samples of H-1 and H-2 vulcanized rubber, respectively.

**Figure 10:**
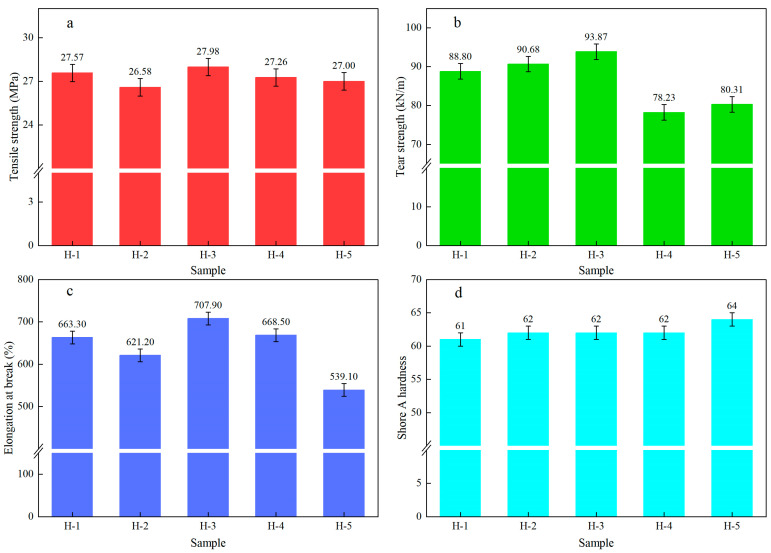
The physical and mechanical properties of natural rubber vulcanized rubber (carbon black formulation). (**a**) Tensile strength; (**b**) Tearing strength; (**c**) Elongation at break; (**d**) Shore A hardness.

The changes in the physical and mechanical properties of natural rubber vulcanized rubber (carbon black formulation) are shown below (Figure 10). As can be seen from the figure, compared with the acid-cured natural rubber vulcanized rubber, the tensile strength and elongation at break of enzyme-assisted microbial coagulation natural rubber vulcanized rubber were relatively low, while the tear strength and shore A hardness were relatively high. This indicates that some of the performances of the acid-coagulated natural rubber vulcanized rubber under the carbon black formulation were better than those of the acid-coagulated natural rubber vulcanized rubber. When the ratio of fresh latex of PR107, Reyan 72059, and Reyan 73397 was 1:1:3, the tensile strength, tear strength, and elongation at break of the natural rubber vulcanized rubber reached the maximum values of 27.98 MPa, 93.87 kN/m, and 707.90%, respectively, indicating that the proper proportioning of fresh latex can significantly improve the physical and mechanical properties of the resulting vulcanized rubber. Compared to the vulcanized rubber properties of natural rubbers under either a carbon black formulation and a pure rubber formulation, the tensile strength and tear strength of acid-coagulated natural rubber vulcanized rubber increased by 4.17 MPa and 64 kN/m, respectively, representing improvements of 17.82% and 258%. Meanwhile, the tensile strength and tear strength of enzyme-assisted microbial-coagulated natural rubber vulcanized rubber increased by up to 1.38 MPa and 62.77 kN/m, representing increases of 5.19% and 202%, respectively. This indicates that the increases in acid-coagulated natural rubber vulcanized rubber are higher than those in other samples, suggesting that carbon black has the best reinforcing effect on acid-coagulated natural rubber vulcanized rubber. This is because acid-coagulated natural rubber has the highest fatty acid content; fatty acids physically soften the rubber, improving the dispersion of carbon black in the rubber [52]. At the same time, the molecular weight and the hardness of the acid-coagulated natural rubber are the smallest, and it is easy to obtain plasticity during the plasticizing process, augmenting the benefits of carbon black dispersed in the natural rubber [53,54].

## 5. Conclusions

In this study, as the ratio of PR107 and Reyan 72059 fresh latex increased, the ash content, volatile matter content, fatty acid content, gel content, and PDI of natural rubber decreased gradually. In contrast, the impurity content, PRI, nitrogen content, Mw, and Mn increased gradually. Under a pure rubber formulation, the ratio of PR107, Reyan 72059, and Reyan 72059 fresh latex were 1:1:3. At this ratio, the tensile strength, tear strength, and modulus at 100% and 300% of the natural rubber vulcanized rubber reached their maximum values, respectively, at 26.60 MPa, 31.10 kN/m, 0.70 MPa, and 1.57 MPa, showing a significant improvement compared to the control sample. Compared to the performance of natural rubber vulcanized rubber under carbon black formulations and pure rubber formulations, the tensile strength of acid-coagulated natural rubber vulcanized rubber shows a much higher increase than that of other natural rubber vulcanized rubbers. This is due to the synergistic effects of fatty acid content and molecular weight in acid-coagulated natural rubber. Compared with the existing literature, this study is the first to systematically analyze the impact of different fresh latex ratios on the composition and properties of natural rubber, clarifying the correspondence between fresh latex ratios and natural rubber properties, and providing technical support for the production and processing of high-performance natural rubber. However, this study lacks research on the structure of natural rubber vulcanized rubber under the pure rubber formulation, making it difficult to provide strong theoretical support for elucidating the mechanisms by which natural rubber composition affects its properties. To address the limitations of the current study, future research will focus on investigating the effects of different fresh latex ratios on the composition, structure, and properties of natural rubber, providing important supplementary insights for theoretical development and technological improvement in this field. This study offers new perspectives for the development of high-performance natural rubber processing technologies. Moving forward, we will refine and optimize the ratios of different fresh latex varieties and select the most suitable fresh latex ratio as raw material for high-performance natural rubber processing, thereby enhancing the overall performance of high-performance natural rubber.

## Figures and Tables

**Table 1:** Ratio and coagulation method of fresh latex.

Sample	Variety	Ratio	Coagulation Method	Enzyme Amount (%, *w*/*w*)	Microbial Amount (%, *w*/*w*)
H-1	73397	0	Acid coagulation	0	0
H-2	73397	0	Enzyme-assisted microbial coagulation	0.05	10
H-3	107:72059:73397	1:1:3	Enzyme-assisted microbial coagulation	0.05	10
H-4	107:72059:73397	1:1:2	Enzyme-assisted microbial coagulation	0.05	10
H-5	107:72059:73397	1:1:1	Enzyme-assisted microbial coagulation	0.05	10

Note: fresh latex ratio, in terms of latex mass; enzyme dosage, in terms of dry gel content; microbial coagulant dosage, in terms of fresh latex mass.

**Table 2:** The pure rubber formulations of natural rubber.

Formulation	Raw Rubber	Stearic Acid	Zinc Oxide	Accelerator M	Sulphur
Mass, g	100	0.5	6.0	0.5	3.5

**Table 3:** The carbon black formulations of natural rubber.

Formulation	Raw Rubber	Zinc Oxide	Sulfur	Stearic Acid	Carbon Black	TBBS ^1^
Mass, g	100	5.0	2.25	2.0	35.00	0.70

^1^ N-tert-butyl-2-benzothiazole sulfonamide, powder form, with ether or ethanol insoluble matter content less than 0.3% (by mass). It should be stored at room temperature in a sealed container and tested for ether or ethanol insoluble matter content every six months. If this content exceeds 0.75% (by mass), the material should be discarded or recrystallised.

**Table 4:** Physical and chemical indexes of natural rubber.

Sample	Ash Content, %	Volatile Matter Content, %	Dirt Content, %	P_0_	PRI, %	M_L(1+4)_
H-1	0.41 ± 0.02	0.37 ± 0.02	0.026 ± 0.004	39.73 ± 0.61	83.70 ± 0.75	81.67 ± 0.58
H-2	0.45 ± 0.03	0.35 ± 0.03	0.025 ± 0.003	45.43 ± 0.65	77.20 ± 0.82	95.00 ± 1.00
H-3	0.43 ± 0.02	0.33 ± 0.03	0.025 ± 0.005	44.97 ± 0.31	77.37 ± 0.4	92.00 ± 0.00
H-4	0.42 ± 0.04	0.32 ± 0.02	0.027 ± 0.005	46.40 ± 0.53	78.50 ± 0.89	93.67 ± 0.58
H-5	0.40 ± 0.03	0.30 ± 0.02	0.027 ± 0.006	46.30 ± 0.36	82.10 ± 0.46	92.67 ± 1.53

**Table 5:** Vulcanization characteristics of natural rubber compound.

Sample	M_L_/dN·m	M_H_/dN·m	ΔM/dN·m	t_10_/min	t_90_/min	CRI/s^−1^
H-1	0.68	5.61	4.93	1.72	19.13	0.17
H-2	0.75	6.52	5.77	1.35	12.82	0.11
H-3	0.73	6.94	6.21	1.28	12.53	0.11
H-4	0.7	6.63	5.93	1.27	12.7	0.11
H-5	0.56	6.61	6.05	1.4	13.18	0.12

Note: ΔM = M_H_ − M_L_; CRI = 100/(t_90_ − t_10_).

## Data Availability

The data presented in this study are available on request from the corresponding author.
